# Nocturnal incubation recess and flushing behavior by duck hens

**DOI:** 10.1002/ece3.7561

**Published:** 2021-05-01

**Authors:** Rebecca Croston, Sarah H. Peterson, C. Alex Hartman, Mark P. Herzog, Cliff L. Feldheim, Michael L. Casazza, Joshua T. Ackerman

**Affiliations:** ^1^ U.S. Geological Survey Western Ecological Research Center Dixon Field Station Dixon CA USA; ^2^ Suisun Marsh Program California Department of Water Resources Sacramento CA USA

**Keywords:** dabbling duck, gadwall, iButton, incubation recess, mallard, nest depredation, nocturnal behavior, nocturnal incubation recess

## Abstract

Incubating birds must balance the needs of their developing embryos with their own physiological needs, and many birds accomplish this by taking periodic breaks from incubation. Mallard (*Anas platyrhynchos*) and gadwall (*Mareca strepera*) hens typically take incubation recesses in the early morning and late afternoon, but recesses can also take place at night. We examined nocturnal incubation recess behavior for mallard and gadwall hens nesting in Suisun Marsh, California, USA, using iButton temperature dataloggers and continuous video monitoring at nests. Fourteen percent of all detected incubation recesses (*N* = 13,708) were nocturnal and took place on 20% of nest‐days (*N* = 8,668). Video monitoring showed that hens covered their eggs with down feathers when they initiated a nocturnal recess themselves as they would a diurnal recess, but they left the eggs uncovered in 94% of the nocturnal recesses in which predators appeared at nests. Thus, determining whether or not eggs were left uncovered during a recess can provide strong indication whether the recess was initiated by the hen (eggs covered) or a predator (eggs uncovered). Because nest temperature decreased more rapidly when eggs were left uncovered versus covered, we were able to characterize eggs during nocturnal incubation recesses as covered or uncovered using nest temperature data. Overall, we predicted that 75% of nocturnal recesses were hen‐initiated recesses (eggs covered) whereas 25% of nocturnal recesses were predator‐initiated recesses (eggs uncovered). Of the predator‐initiated nocturnal recesses, 56% were accompanied by evidence of depredation at the nest during the subsequent nest monitoring visit. Hen‐initiated nocturnal recesses began later in the night (closer to morning) and were shorter than predator‐initiated nocturnal recesses. Our results indicate that nocturnal incubation recesses occur regularly (14% of all recesses) and, similar to diurnal recesses, most nocturnal recesses (75%) are initiated by the hen rather than an approaching predator.

## Graphical abstract

We examined nocturnal incubation recess behavior for mallard and gadwall hens using iButton temperature dataloggers and continuous video monitoring at nests. Fourteen percent of all detected incubation recesses (*N* = 13,708) were nocturnal and took place on 20% of nest‐days (*N* = 8,668). Using the observation that nest temperature decreased more rapidly when eggs were left uncovered versus covered, we developed a method to characterize eggs during nocturnal incubation recesses as covered or uncovered using nest temperature data. Overall, we predicted that 75% of nocturnal recesses were normal, hen‐initiated recesses (eggs covered) whereas 25% of nocturnal recesses were predator‐initiated recesses (eggs uncovered).

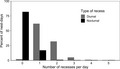

## 1 INTRODUCTION

During incubation, most birds must balance the needs of their developing embryos with their own physiological needs (Reid et al., 2002; Tinbergen & Williams, 2002). In dabbling ducks, as with many other birds, maintaining consistent egg temperatures throughout incubation is energetically expensive, so hens must take periodic breaks from incubation to forage and replenish resources (Tinbergen & Williams, 2002). Typically mallard (*Anas platyrhynchos*) and gadwall (*Mareca strepera*) hens take recesses from incubation once or twice per day, in the early morning and the late afternoon (Croston et al., 2020; Hoover, 2002; Lorenz, 2005). However, incubation recesses can also occur at night, whether initiated by the hen as a normal recess, or initiated when a nearby predator caused the hen to flush from the nest.

Duck nest depredation, particularly by mammals, occurs predominantly at night (e.g., Croston, Ackerman, et al., 2018; Lariviere & Messier, 2001). Mammalian predators accounted for 71% of nest depredation events and 89% of eggs depredated within Grizzly Island Wildlife Area, Suisun Marsh, CA, USA, with most depredation events occurring between 20:00 and 04:00 hr (Croston, Ackerman, et al., 2018). During most nest depredation events, hens left the nest immediately (on average 29 s) before the predator appeared at the nest (Croston, Ackerman, et al., 2018).

Hens may also voluntarily leave the nest to forage and drink at night as they would during a normal diurnal incubation recess. Incubation rhythms are typically flexible and are responsive to both physiological (e.g., Cooper & Voss, 2013; Criscuolo et al., 2002) and environmental factors (e.g., Afton, 1980; Coates et al., 2016; Ringelman et al., 1982). Nocturnal incubation recesses may be necessary if hens cannot adequately meet their needs during daytime recesses, or to avoid competition or exploit increased prey availability at night (McNeil et al., 1992). Alternatively, hens may leave nests unattended at night when the risk of depredation is high to avoid risk to themselves and protect against the loss of future reproductive efforts (Cervencl et al., 2011). Studies of nocturnal activity in waterfowl have shown a variety of behaviors (Aldrich & Raveling, 1983; Ebbinge et al., 1975; Madsen et al., 1989; Moulton & Weller, 1984; Paulus, 1984; Pedroli, 1982; Raveling et al., 1972; Tamisier, 1976), but few studies have quantified nocturnal behaviors of dabbling ducks during incubation.

We examined the frequency, timing, and duration of nocturnal incubation recesses in dabbling ducks, and the causes of these nocturnal recesses. Using video camera data to ground‐truth events at the nest, we developed and tested a technique that uses nest temperature data to identify and differentiate normal nocturnal recesses initiated by hens from nocturnal recesses that were likely triggered by predators. Specifically, we took advantage of hens’ typical behavior of covering eggs with down feathers and other nest materials before leaving the nest when they initiated incubation recesses. Conversely, when approaching predators caused hens to flush from their nests suddenly, they typically did not cover their eggs (Croston, Ackerman, et al., 2018). Combining this observation with corresponding differences in the rate of nest cooling once hens left their nests, we differentiated normal, hen‐initiated incubation recesses from recesses occurring when hens flushed from their nests, likely in response to predators nearby.

## 2 METHODS

We monitored dabbling duck nests at the Grizzly Island Wildlife Area, Suisun Marsh, CA, USA, during breeding seasons 2015–2018. We located nests by systematically searching upland fields using standard nest searching methods modified from McLandress et al. (1996). We flushed incubating hens from nests by dragging a 50‐m rope strung between two all‐terrain vehicles across the tops of vegetation and marked nests with a 2‐m bamboo stake placed 4‐m north of the nest and a vegetation‐height stake placed on the southern rim of the nest bowl. We candled eggs (sensu Weller, 1956) to track embryonic development and to determine the incubation stage at which the nest was found. To estimate the date of clutch completion for nests found during laying, we counted forward assuming that one egg was laid per nest‐day. For nests found during incubation, we subtracted the average incubation stage of all eggs at the first visit from the date of the first visit to estimate the date of clutch completion. We revisited nests approximately weekly until either hatching or failure and documented any evidence of depredation (e.g., missing eggs, broken eggshells) since the previous nest visit.

We monitored nest temperature using iButton™ temperature dataloggers (Model DS1922L‐F5#, Maxim Integrated Products, Inc.) placed within each nest bowl and identified incubation recesses based on monotonic decreases in nest temperature relative to each individual nest's daily variation in incubation temperature following methods detailed in Croston et al. (2018). Prior to placement in nests, we inserted iButtons into and protruding slightly above the tops of cream‐colored rubber stoppers and affixed these to long nails, which allowed us to anchor the iButtons firmly into the ground and flush with the apical surface of the eggs. We programmed iButtons to record temperatures every 4 min in 2015 and every 8 min in 2016–2018. Temperature data in 2015 were censored every 8 min for direct comparison with 2016–2018 data. In 2015, we replaced iButtons every two weeks to prevent onboard memory from becoming full (the longer 8‐min interval used in 2016–2018 allowed a single iButton to collect data over the lifetime of a nest). At each nest, we placed one iButton in the nest bowl among the eggs, and a second iButton just outside of the southern rim of the nest bowl to record ambient temperature at the nest. We excluded from analyses any data collected on days that investigators visited nests (from nautical dawn on the visit day through nautical dawn on the following calendar day), except in the case of assessing rate of temperature decrease during individual recesses (see below). We also excluded any data collected at nests where researchers visited more than 5 times (*N* = 57 nests) and any data collected on nest‐days with >5 recesses (an additional 16 nest‐days), as these likely resulted from prolonged poor contact between the iButton and the hen's brood patch. Diurnal recesses were defined as having started between nautical dawn and nautical dusk each day, and nocturnal recesses were defined as having started between nautical dusk and the following nautical dawn. For frequency analyses, each day was defined as nautical dawn through the following nautical dawn.

During the 2015–2017 breeding seasons, we simultaneously monitored a subset of nests at >6 days incubation with continuous video recording using compact cylindrical cameras (EZspy Cam; IR Nightvision Camera #ENC‐102NRA, Day Night Lipstick Camera with invisible 950‐nm infrared LED and 3.6‐mm lens) mounted directly above the nest bowl, allowing for a viewable area of approximately 0.5 m^2^ which included the nest bowl and its immediate periphery. The camera battery and digital video recorder (DVR) were placed approximately 50 m from nests in order to limit nest disturbance, and we downloaded data and replaced camera batteries during weekly nest visits.

We associated nocturnal recesses identified with nest temperature data with coincident data collected from nest cameras and viewed the associated video footage (including 10 min prior and 30 min after the start of the recess identified with nest temperature data) to verify that the hen left the nest, determine whether or not the hen covered the eggs prior to leaving the nest, and determine whether and what (if any) predator appeared at the nest. We analyzed data from both cameras and iButtons that were collected at nests with completed clutches and only while nests remained active (as assessed through visual examination of temperature data). This means that for cases in which hens left the nest and did not return, whether because of depredation or abandonment, the start time of that recess is recorded and is included in analysis of recess frequency, but there is no recess end time or duration associated with that recess and therefore it is not included in analyses with recess duration. For nests that ultimately hatched, we excluded data from the full day prior to the last day a nest was active, because investigators made additional visits to the nest at this time.

### 2.1 Statistical analysis

#### 2.1.1 Nocturnal versus diurnal recess frequency and duration

We examined the frequency and duration of nocturnal recesses using nest temperature data and linear mixed models (LMMs, R package *lme4*, Bates et el., 2019) with restricted maximum likelihood, and with Type III Wald *F* tests and Kenward‐Roger degrees of freedom (R package *car*, Fox & Weisberg, 2019). All models were fitted with Gaussian error distribution, and variables were transformed to improve normality where noted below. We present summary results both as raw data and as model predicted medians and 95% prediction intervals (2.5th and 97.5th quantiles) bootstrapped over 1,000 iterations.

We first compared the frequencies of nocturnal versus diurnal recesses. To correct for the difference in length between the diurnal and nocturnal periods each nest‐day, we modeled recess frequency as number of recesses per nest‐hour by dividing the number of each type of recess per nest‐day by the number of daylight or night (as applicable) hours in that day to get an hourly recess rate for each day and night for each type. We fit an LMM with the number of recesses per nest‐hour as the response variable, and with the type of recess (nocturnal/diurnal) and its interaction with species as fixed effects and nest identification as a random effect. We did not include incubation day or ambient temperature at the nest as covariates in the model, because the effect sizes associated with these parameters were very small, even across the entire incubation period (Croston et al., 2020).

Next, we compared the duration of nocturnal recesses (recess end time–recess start time adjusted for lag in detection of hen departure and return; Croston, Hartman, et al., 2018) with the duration of diurnal recesses. We fit an LMM with natural log‐transformed recess duration as the response variable, with type of recess (nocturnal and diurnal), species (gadwall and mallard), and their interaction as categorical fixed effects, and with nest identification as a random effect.

#### 2.1.2 Characterizing nocturnal recesses using video data

Punctuated monotonic decreases in nest temperature can be used to determine whether and when hens stop incubating their eggs, including at night (Croston, Hartman, et al., 2018). However, punctuated monotonic decreases in nest temperature alone do not allow us to ascertain whether a hen initiated a recess on her own or was flushed from the nest by a predator. The rate of nest temperature change during a nocturnal recess may help differentiate between these two scenarios. Dabbling duck hens typically cover their eggs with down and other nest material prior to leaving nests when they initiate an incubation recess (e.g., Ackerman et al., 2003), but do not when they are flushed from their nests by approaching predators (e.g., Croston, Ackerman, et al., 2018). Moreover, we observed that only 6% of nest depredations observed on video occurred when eggs were covered, whereas 94% of nest depredations occurred when eggs were not covered (Table 1), and hens did not flush from the nest when a predator approached until an average of 29 s before the predator arrived at the nest (Croston, Ackerman, et al., 2018). Therefore, covered eggs are a good indication that the hen initiated a normal incubation recess rather than being flushed from the nest by an approaching predator (hereafter: predator‐initiated recess), and if a nest was depredated any time between investigators’ nest monitoring visits, then a nocturnal recess in which eggs were not covered that took place within that time period is likely to represent a predator‐initiated recess. Thus, if the rate of nest temperature change differs when eggs are covered compared with when eggs are uncovered, investigators may be able to differentiate types of nocturnal incubation recesses using nest temperature data alone.

**TABLE 1:** Number of nocturnal recesses at nests with small video cameras during which hens left the nest with eggs covered versus not covered and whether a predator was observed on camera at the nest. Note that only 6% (2 out of 34) of the recesses in which a predator was observed at the nest occurred after hens had covered their eggs

	Eggs covered with nest material	Eggs not covered with nest material
Predator observed at nest	2	32
No predator observed at nest	144	29 (+1^a^)

^a^ In one case, a hen removed her own egg from the nest.

Using nocturnal recesses for which we had both video and nest temperature data, we investigated whether the rate of temperature decrease during a recess could be used to determine whether eggs were covered or left uncovered by the hen when she left for an incubation recess, and therefore, if the rate of nest temperature decrease could be used to identify recesses that likely resulted from predators approaching nests and the hen flushing from the nest without covering the eggs. To identify and differentiate these types of nocturnal incubation recesses, we used a 4‐step approach. First, we calculated the rate of decrease in nest temperature during the first 32 min of each recess. Standardizing the time interval after recesses began allowed us to calculate the rate of temperature decrease while controlling for differences in nest temperature that resulted from differences in total recess duration. Second, we modeled differences in the rate of nest temperature decrease during recesses in which eggs were covered versus uncovered as determined by video monitoring data. This model controlled for both nest temperature and ambient temperature at the start of the incubation recess and included nest identification as a random effect. In this and all models, rate of nest temperature decrease was natural log‐transformed to improve normality. Third, we fit a binomial GLMM predicting the probability that nests were covered as a function of the rate of nest temperature decrease. We accounted for nest and ambient temperature at the start of the recess by including them as covariates in the model and included nest identification as a random effect. Fourth, we used the fitted binomial model to generate the predicted probability that eggs were covered during each actual recess in the dataset, and combined this with the physical status of the nest found during the next nest monitoring visit (evidence versus no evidence of depredation) to predict whether predators did or did not appear at nests during each nocturnal recess. If the predicted probability that eggs were covered was >50% and we did not find evidence of depredation at the next nest monitoring visit, we assigned that recess “covered eggs with no depredation.” If the predicted probability that eggs were covered was >50%, and we found evidence of depredation at the next nest monitoring visit, we assigned that recess “covered eggs with depredation.” If the predicted probability that eggs were covered was ≤50%, and we did not find evidence of depredation at the next nest monitoring visit, we assigned that recess “uncovered eggs with no depredation.” Finally, if the predicted probability that eggs were covered was ≤50%, and we found evidence of depredation at the next nest visit, we assigned that recess “uncovered eggs with depredation.” We tested this approach with a subset of our data by comparing our predicted assignments with events that were actually observed on video.

#### 2.1.3 Predicting type of nocturnal recess using nest temperature change

Once we determined that this approach could be used to differentiate among types of nocturnal recesses, we applied it to our larger dataset consisting of nest temperature data from the breeding seasons 2015–2018. We assigned each nocturnal recess in the full nest temperature dataset into one of four categories based on whether we predicted the nest was covered and whether or not we found eggs missing or damaged at the next nest monitoring visit, as described above, except that for the full dataset we extended our predictions (covered/uncovered) to include recesses that were shorter than 32 min, by dividing the temperature difference by the duration of the recess to calculate an initial rate of temperature decrease. We used LMMs to compare the (a) start time and (b) duration of all recesses among all four types of nocturnal recess. Both of these models included the predicted recess type as a fixed effect, with incubation day and species. Both models also included nest identification as a random effect.

## 3 RESULTS

### 3.1 Nocturnal versus diurnal recess frequency and duration

We detected 1,898 nocturnal recesses at 521 nests and 11,810 diurnal recesses at 779 nests over 8,668 nest‐days during the 2015–2018 breeding seasons using iButton temperature data (Croston et al., 2021). In total, 14% of all incubation recesses that we detected were nocturnal, and 67% of all nests had at least one nocturnal recess. Hens took 1.36 ± 0.61 diurnal recesses per nest‐day (mean ± *SD*), and 0.22 ± 0.13 nocturnal recesses per nest‐day. Among nest‐days that had ≥1 nocturnal recess (i.e., excluding nights with 0 recesses), hens took 1.07 ± 0.28 nocturnal recesses per nest‐day.

**FIGURE 1:**
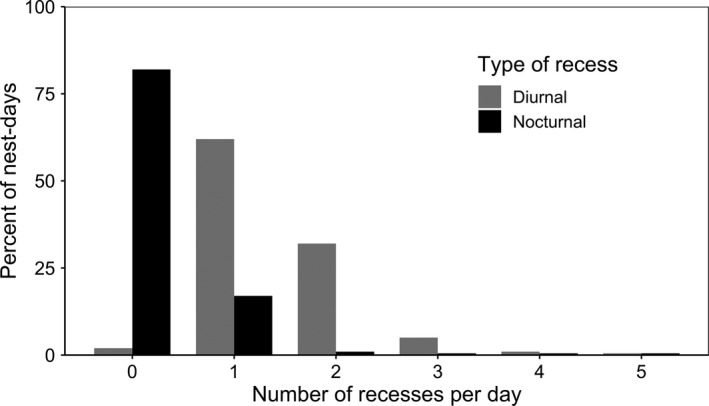
Percent of all nest‐days (24‐hr periods at each nest) with 0 to 5 diurnal (gray bars) or nocturnal (black bars) incubation recesses for mallard and gadwall hens nesting in Grizzly Island Wildlife Area, Suisun Marsh, CA, USA, during 2015–2018. Note that only 19% of nest‐days had any nocturnal recesses and that 94% of nest‐days that had at least one nocturnal recess had only one nocturnal recess

**FIGURE 2:**
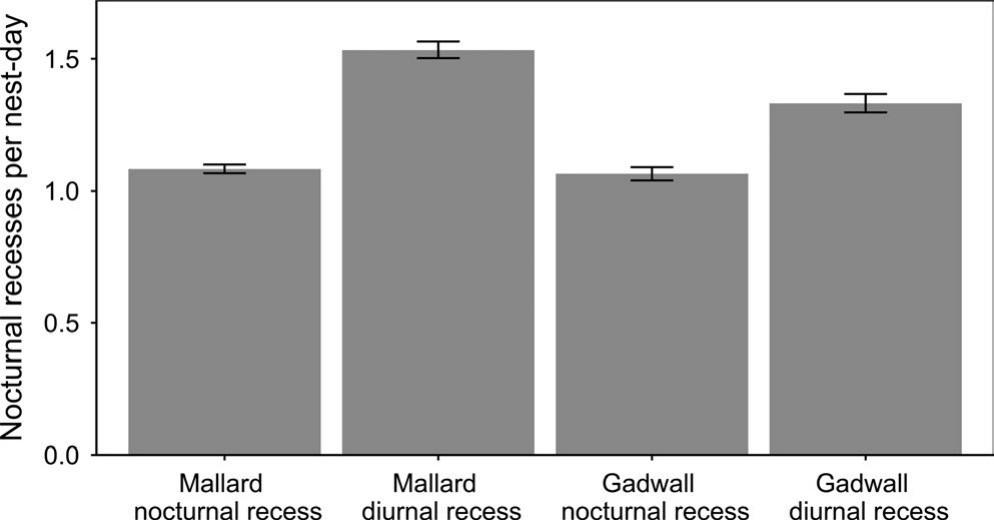
Predicted frequency per nest‐day for nest‐days with ≥1 one nocturnal or diurnal recess for mallard and gadwall nesting in Grizzly Island Wildlife Area, Suisun Marsh, CA, USA, during 2015–2018. On average, daylight lasted 17.13 hr and night lasted 6.87 hr. Note that only 19% of nest‐days had any nocturnal recesses

Most nest‐days were characterized by one (62% of nest‐days) or two (32%) diurnal recesses, and zero (82%) or one (17%) nocturnal recess (Figure 1). Both mallard and gadwall took significantly more diurnal recesses than nocturnal recesses per day (*F*_1,9,943.40_ = 1865.61, *p* <.0001; Figure 2), but this difference was greater for mallard than for gadwall (type of recess ×species *F*_1,9,954.70_ = 18.06, *p* <.0001). Likewise, the predicted duration (median [95% prediction interval]) of recesses differed between nocturnal and diurnal recesses (*F*_1,13,466.05_ = 47.99, *p* <.0001), and the relationship between recess duration and type of recess differed among species (type of recess × species *F*_1,13,442.80_ = 6.21, *p* <.05; mallard nocturnal recesses: 122.98 [117.65; 128.70]) min; mallard diurnal recesses: 141.66 [137.81; 146.07] min; gadwall nocturnal recesses: 132.05 [122.46; 141.55] min; and gadwall diurnal recesses: 168.99 [163.46; 174.80] min).

### 3.2 Characterizing nocturnal recesses using video data

We detected 224 nocturnal recesses using iButton temperature data at 72 unique nests that were also monitored with continuous video recording. During 7% (*N* = 16) of events classified with nest temperature data (based on monotonic changes in nest temperature relative to each nest's daily variation in nest temperature; sensu Croston, Ackerman, et al., 2018) as nocturnal recesses, the camera documented that the hens actually remained at their nests (hereafter: false positives). In seven of these 16 cases, the hen stood up and/or adjusted the eggs on camera. In one case, the hen was disrupted by a gopher snake (*Pituophis catenifer*) during an attempted egg depredation event but did not leave the nest. During the other eight false positives in automated recess detection, hens simply continued to incubate eggs without any observed disruption. These eight cases most likely represented situations in which the iButton was not in contact with the hens’ brood patch, resulting in a temperature decrease despite continued incubation by hens. All 16 cases were removed from further analysis of events observed on camera as there were no corresponding recess information available in the video data for these events (as there were no recesses), but they were not removed from the full data analyses as we would not have known that these data were not accurate if we only had nest temperature data available to define these events.

The remaining 208 nocturnal incubation recesses were confirmed with video monitoring data. Of these, 75% (*N* = 157) were nocturnal recesses in which hens left the nest, no predator appeared at the nest, and we found no evidence of depredation during the next nest monitoring visit (*N* = 132 recesses with covered eggs and *N* = 25 with uncovered eggs). An additional 14% (*N* = 30) of the nocturnal recesses had predators enter the camera frame while hens were away, and we found evidence of depredation during the next nest monitoring visit (*N* = 28 recesses with uncovered eggs and *N* = 2 with covered eggs). Two percent (*N* = 4) were predator‐initiated recesses in which a predator appeared in the camera frame and flushed the hen off the nest, but we found no evidence of depredation at the next nest visit. One of these events occurred when a Tule elk (*Cervus canadensis nannodes*) approached the nest, one involved a gopher snake, one involved a rat (*Rattus* sp.), and one involved a striped skunk (*Mephitis mephitis*) which did not depredate any eggs. In the remaining 8% of cases (*N* = 17), a nocturnal recess occurred and we found evidence of nest depredation during the subsequent nest visit, but no predator was observed on camera during that specific recess. Of these, the eggs were covered during 12 nocturnal recesses and were left uncovered during four nocturnal recesses. In the remaining case, the hen removed her own egg from the nest, and we removed this event from further analysis. Without the video camera data, these 17 nocturnal recesses would have been incorrectly identified as predator‐initiated recesses associated with depredation at the nest.

### 3.3 Predicting type of nocturnal recess using nest temperature change

Using the nocturnal incubation recesses that we detected with temperature dataloggers and confirmed with continuous video monitoring, we investigated differences in the rate of nest temperature change during nocturnal recesses with covered versus uncovered eggs. Nest temperature decreased more slowly during nocturnal recesses when eggs were covered than when eggs were not covered (0.07 [0.07; 0.08] °C/min covered, 0.21 [0.19; 0.25] °C/min not covered; *F*_1,161.68_ = 139.86, *p* < .0001), after accounting for effects of ambient temperature (*F*_1,164.79_ = 3.42, *p* =.07) and nest temperature (*F*_1,163.63_ = 26.62, *p* < .0001) at the beginning of the recess.

**FIGURE 3:**
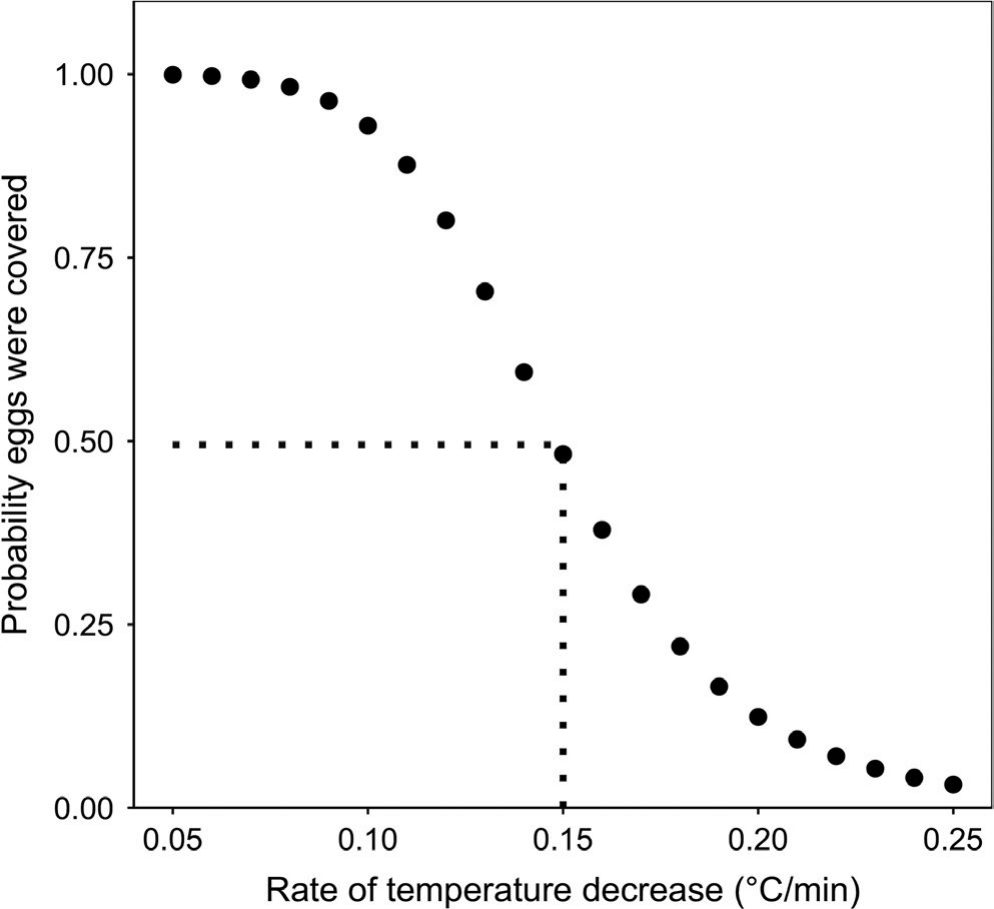
The predicted probabilities of nests being covered with down feathers during an incubation recess for mallard and gadwall hens nesting at Grizzly Island Wildlife Area, Suisun Marsh, CA, USA, in 2015–2018. In this example, where initial ambient temperature and initial temperature were set at median values of the data, a 50% probability (dotted horizontal line) of the eggs being covered with down feathers occurred at a nest temperature decrease rate of 0.155°C/min (dotted vertical line). For each incubation recess, we used recess‐specific covariate values (rate of nest temperature decrease, starting ambient temperature, starting nest temperature) to predict the probability that the eggs were covered, and assigned recesses with a ≤50% probability as being uncovered and >50% as being covered

We found that the rate of temperature decrease predicted whether or not eggs were covered during each recess (*χ* ^2^ = 14.34, *df* = 1, *p* < .0005) after accounting for initial ambient temperature (*χ* ^2^ = 9.64, *df* = 1, *p <* .005) and initial nest temperature (*χ* ^2^ = 15.34, *df* = 1, *p* < .0005) as covariates. From our model fit (Figure 3), we generated predicted probabilities that eggs were covered during each actual nocturnal incubation recess in our dataset.

The likelihood of correctly identifying nonpredator events (in which hens left the nest but no predator appeared on camera, whether or not eggs were covered) was dependent in part on whether or not we incorporated the status of the nest at our next monitoring visit into our assessment of events. This is because most of our errors resulted from recesses during which eggs were left uncovered but no eggs were damaged or removed, either because a predator was nearby but did not find the nest or because a predator found the nest but did not depredate any eggs (as is the case with gopher snakes; Croston, Ackerman, et al., 2018). Using only the predicted probability that eggs were uncovered from the binomial model (*N* = 175 total recesses), we correctly predicted predator presence in 93% (*N* = 27 of 29) of cases and no predator presence in 88% (*N* = 129 of 146) of cases. When we also incorporated the status of the nest at the next nest visit into our assessment, we correctly predicted the specific recesses in which predators appeared at nests in 90% (*N* = 26 of 29) of cases and the specific recesses in which no predator appeared at the nest in 98% (*N* = 143 of 146) of cases.

### 3.4 Frequency and timing of nocturnal recesses based on nest temperature data (all nests)

We predicted the probability that eggs were covered for 1,794 nocturnal recesses (104 nocturnal recesses were missing ambient temperature data and were removed from the dataset because the probability that eggs were covered could not be predicted). We combined this with the status of the nest at the next nest monitoring visit (whether or not we found evidence of nest depredation) in order to categorize recesses as one of the four types described in Section 2: (a) covered eggs with no depredation, (b) covered eggs with depredation, (c) uncovered eggs with no depredation, and (d) uncovered eggs with depredation.

In 63% of recesses (*N* = 1,125 of 1,794) eggs were predicted to be covered and we found no evidence of depredation at the nest during the following nest monitoring visit (covered eggs with no depredation). In 12% of recesses (*N* = 215), eggs were predicted to be covered and were accompanied by evidence of depredation (covered eggs with depredation). In 11% of recesses (*N* = 202), eggs were predicted to be uncovered and we found no evidence of depredation (uncovered eggs with no depredation), and in another 14% (*N* = 252), eggs were predicted to be uncovered and the recess was accompanied by evidence of depredation at the next nest monitoring visit (uncovered eggs with depredation).

We found a limited number of nocturnal recesses in which eggs were uncovered within nest monitoring intervals where evidence of depredation was observed, and this allowed us to deduce specifically when predator activity occurred at the nest. In fact, 79% (*N* = 156 of 198) of all nest monitoring intervals in which we found evidence of depredation had only a single nocturnal recess that was categorized as uncovered within that interval. Another 17% had two nocturnal recesses with uncovered eggs, and the remaining 4% had three to five.

**FIGURE 4:**
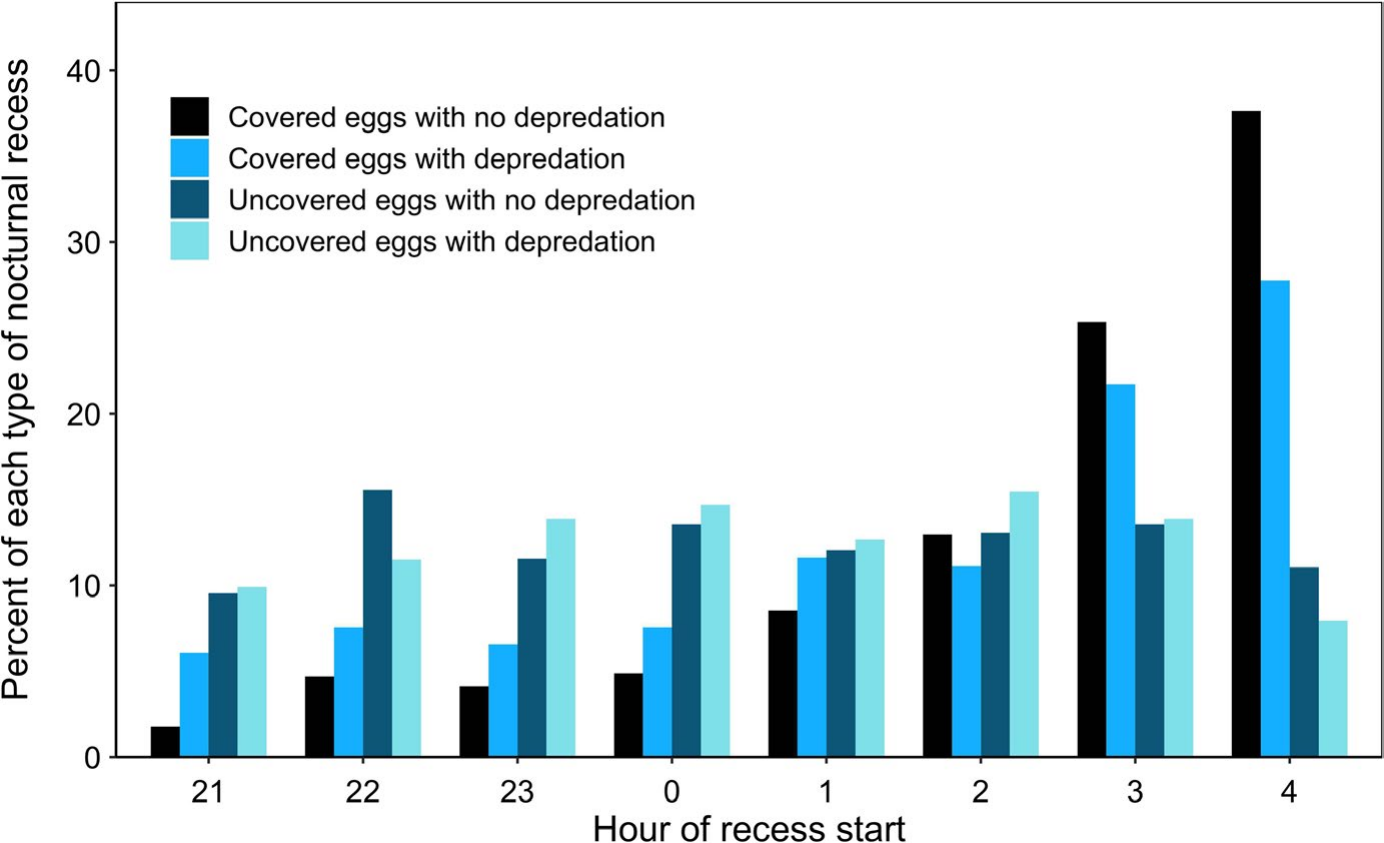
Hour of start times for nocturnal incubation recesses in which eggs were covered and we found no evidence of depredation (64% of nocturnal incubation recesses), eggs were covered and we found evidence of depredation (11%), eggs were uncovered and we found no evidence of depredation (11%), and eggs were uncovered and we found evidence of depredation (14%) as determined by the probability that eggs were covered during an incubation recess and the observed nest status at the next nest monitoring visit, for mallard and gadwall hens nesting within Grizzly Island Wildlife Area, Suisun Marsh, CA, USA, during breeding seasons 2015–2018

**FIGURE 5:**
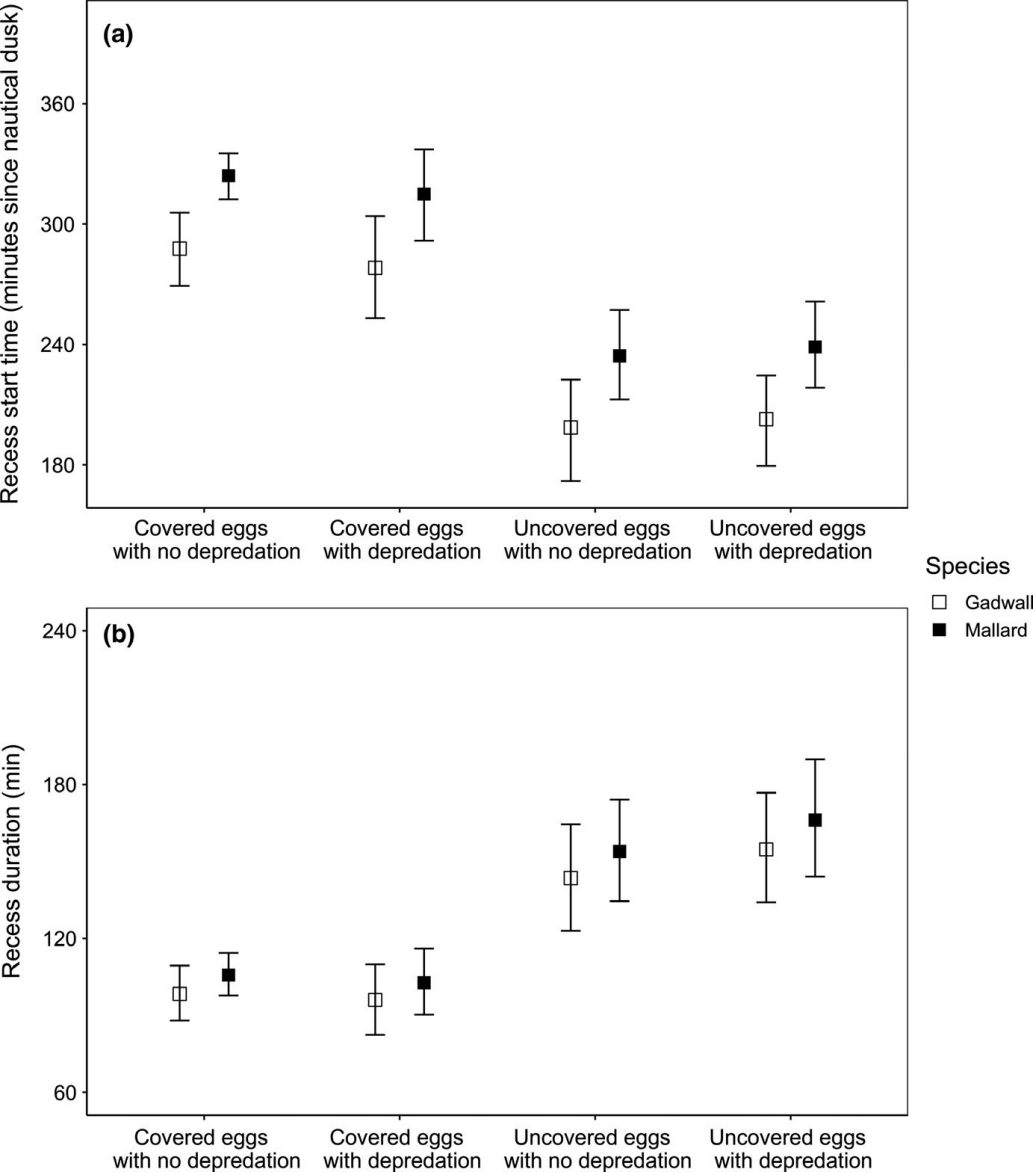
Predicted median a) start times and b) durations of nocturnal incubation recesses in which eggs were covered and we found no evidence of depredation during the subsequent nest monitoring visit, eggs were covered and we found evidence of depredation, eggs were uncovered and we found no evidence of depredation, and eggs were uncovered and we found evidence of depredation, for mallard and gadwall hens nesting within Grizzly Island Wildlife Area, Suisun Marsh, CA, USA, during breeding seasons 2015–2018. Bars represent 95% prediction interval. Predictions are shown with incubation day held constant at day 13. On average, nautical dawn occurred at 04:51 and nautical dusk occurred at 21:25

Model‐predicted recess start times were significantly earlier in the night for recesses categorized as uncovered eggs (with and without evidence of depredation) than for recesses categorized as covered eggs (with and without evidence of depredation; *F*_3,1,403.49_ = 31.21, *p* < .0001; Figures 4 and 5a), after accounting for differences between species (*F*_1,450.33_ = 13.18, *p* < .0005) and effects of incubation day (*F*_1,1,307.79_ = 0.18, *p* = .67). Recesses during which eggs were covered and we found no evidence of depredation occurred more frequently later in the night (closer to dawn) than in earlier parts of the night, while start times for recesses during which eggs were not covered were more evenly distributed throughout the night (Figure 4).

The longest nocturnal recesses occurred when eggs were uncovered and we found evidence of depredation during the next nest monitoring visit. After accounting for decreasing recess duration as incubation progressed (*F*_1,1656.23_ = 22.80, *p* < .0001) and species (*F*_1,485.29_ = 1.14, *p* = .29), we found a significant difference in recess duration among the recess types (*F*_3,1614.37_ = 29.04, *p* < .0001; Figure 5b), primarily the result of the longer recess duration of nocturnal recesses categorized as uncovered eggs with depredation. Nocturnal recesses categorized as uncovered eggs with depredation were 57% longer for mallard and 58% longer for gadwall than recesses in which eggs were covered and we found no evidence of depredation, 8% longer than recesses in which eggs were uncovered and we found no evidence of depredation for both mallard and gadwall, and 61% longer than recesses in which eggs were covered and we found evidence of depredation for both mallard and gadwall.

## 4 DISCUSSION

Maintaining consistent egg temperatures throughout incubation is energetically expensive, and hens must take periodic breaks in order to meet their physiological needs. We found that incubation recesses are not exclusive to daylight time periods. In fact, 16% of all incubation recesses occurred at night between nautical dusk and nautical dawn, and 20% of nest‐days had at least one nocturnal incubation recess. To our knowledge, nocturnal incubation recesses, particularly those initiated by hens, have not been characterized previously for waterfowl, and researchers have generally believed that nesting waterfowl engage in limited nonincubation activities at night (Jorde & Owen, 1988). Hampton (1981) documented regular nightly incubation recesses in nesting trumpeter swans (*Cygnus buccinator*); however, incubation rhythms in these data may have resulted from inaccuracies associated with battery drawdown in the monitoring system (Cooper & Afton, 1981; Henson & Cooper, 1994).

Both mallard and gadwall took substantially more diurnal incubation recesses (1.4 per nest‐day) than nocturnal incubation recesses (0.2 per nest‐day). This overall difference in number of recesses per day was greater for mallard than for gadwall, but the number of nocturnal recesses did not differ between species. Thus, this difference between species is a result of mallards taking more diurnal recesses on average than gadwall (Croston et al., 2020), and not due to differences in nocturnal recess frequency between species. In addition, nocturnal recesses in which eggs were left uncovered were longer than those in which eggs were covered, further suggesting that these nocturnal recesses with covered eggs were initiated by hens and were similar to diurnal recesses. In contrast, hens took longer recesses when nests were likely being depredated (uncovered eggs with evidence of depredation at the subsequent nest visit).

Nocturnal incubation recesses in which eggs were uncovered (25% of nocturnal recesses), whether or not we found evidence of depredation at the next nest monitoring visit, were likely the result of predator activity near the nest, as hens tend to remain on their nests until the predator is near. On average, predators appeared at nests 29 s after hens flushed without covering their eggs (Croston, Ackerman, et al., 2018). Thus, nocturnal recesses in which the eggs were left uncovered and we found evidence of depredation at the next nest monitoring visit likely represented an actual depredation event, in which predators found nests and damaged or displaced eggs. This was further evidenced by the fact that a predator at the nest (observed on camera) was almost always associated with a recess in which hens left eggs uncovered (93% of predator visits), and during a nest‐week that we found evidence of depredation, very few nocturnal recesses with eggs uncovered occurred (79% of nest‐weeks with evidence of depredation had only one nocturnal recess with uncovered eggs). Together, these allowed us to determine with high confidence that a nocturnal recess with uncovered eggs represented the actual date and time that a depredation event occurred during a week that we found evidence of nest depredation. However, it remains possible that any particular recess during which eggs were uncovered was an event in which a predator flushed the hen from her nest but did not depredate it, and the nest was depredated at yet another time between the same two nest monitoring visits. In turn, when a nocturnal recess occurred during which the eggs were left uncovered and we later found no evidence of a nest depredation, a predator likely flushed the hen from her nest but did not find the nest and successfully consume or remove eggs. Examples included nests visited by elk, rats, and snakes, all of which flushed the hens but did not successfully consume or remove eggs.

In the remaining 75% of nocturnal incubation recesses, hens covered their eggs with nest material before leaving the nest, suggesting that the hens initiated the incubation recesses without predator influence, as generally occurs during a normal diurnal incubation recess. It remains possible that these recesses represented responses to predators in the vicinity of the nest and that hens sometimes detected a predator with enough time to cover the eggs before leaving the nest, and thus evade detection by the predator. However, hens typically remain at the nest until immediately before predators arrive (Croston, Ackerman, et al., 2018), and our video data in the present study showed infrequent (1.4%) predator presence at any covered nest, suggesting that hens rarely cover their eggs before leaving the nest as predators approach.

Incubation recesses in which eggs were uncovered and which were associated with evidence of depredation observed during the next nest monitoring visit began earlier in the night on average than either type of nocturnal recess in which hens covered eggs (hen‐initiated) before leaving the nest. This is likely because the timing of depredation events was relatively evenly distributed throughout the night (Figure 4) when mammalian predators are most active (Croston, Ackerman, et al., 2018), whereas hens initiated nocturnal recesses (covered eggs, no evidence of depredation) more frequently later in the night and closer to their first diurnal recess around dawn (mallard: 05:57, gadwall: 05:44; Croston et al., 2020), possibly as a substitute for these dawn recesses for hens that are particularly resource‐depleted.

Incubation recesses in which eggs were uncovered and which were associated with evidence of depredation during the next nest monitoring visit were also significantly longer than any other type of recess (mallard: 166 min; gadwall: 155 min; Figure 5b). The increased duration of incubation recesses initiated by predators may have been because hens avoided returning to nests while predators were active nearby, as evidenced by the difference in latency to return to the nest when flushed by different predators (Croston, Ackerman, et al., 2018). For example, hens take a longer time to return to the nest after being flushed by raccoons (*Procyon lotor*, 239 min) in particular, compared with striped skunks (81 min) or snakes (15 min; Croston, Ackerman, et al., 2018). In fact, hens may stay away from the nest for longer periods of time during partial clutch depredation events to protect themselves and their future reproductive efforts at the expense of increased risk to their current reproductive investment (McNeil et al., 1992, e.g., Cervencl et al., 2011).

Although incubation recesses have been widely understood to provide time for self‐maintenance activities, few studies have identified or investigated nocturnal incubation recesses in detail for waterfowl. We found that nocturnal recesses accounted for 14% of all incubation recesses and that 75% of these nocturnal recesses were likely normal, hen‐initiated recesses whereas only 25% were likely predator‐initiated recesses. This nocturnal recess behavior has direct implications for nest survival as most nest depredation in dabbling ducks occurs at night. Further study could explore the physiological and ecological drivers of nocturnal incubation recess behavior, in particular the relationship between this behavior and hen body condition, and overall predation risk in the population.

## CONFLICT OF INTEREST

The authors declare no conflict of interest.

## AUTHOR CONTRIBUTIONS

**Rebecca Croston:** Conceptualization (lead); Data curation (equal); Formal analysis (equal); Investigation (equal); Methodology (equal); Visualization (equal); Writing‐original draft (lead). **Sarah H Peterson:** Conceptualization (lead); Data curation (equal); Formal analysis (equal); Methodology (equal); Writing‐review & editing (equal). **C. Alex Hartman:** Conceptualization (equal); Data curation (equal); Formal analysis (equal); Methodology (equal); Writing‐review & editing (equal). **Mark P Herzog:** Conceptualization (equal); Data curation (equal); Formal analysis (equal); Methodology (equal); Writing‐review & editing (equal). **Cliff L Feldheim:** Project administration (equal); Resources (equal). **Michael L Casazza:** Conceptualization (equal); Methodology (equal); Project administration (equal); Resources (equal). **Joshua T Ackerman:** Conceptualization (equal); Data curation (equal); Methodology (equal); Project administration (equal); Resources (equal); Writing‐original draft (equal); Writing‐review & editing (equal).

## ETHICS STATEMENT

Research was conducted with the approval of the U.S. Geological Survey Western Ecological Research Center's Animal Care and Use Committee.

## Data Availability

The raw data in this manuscript will be deposited in ScienceBase and are available at https://doi.org/10.5066/P9XG4KSK.
